# A Protocol for the Non-invasive Method of Ultrasound Separation During the Sociosexual Vocal-Non-contact Model in Rats

**DOI:** 10.3389/fnbeh.2022.910591

**Published:** 2022-05-25

**Authors:** Wiktor Bogacki-Rychlik, Anna Wrona, Michal Bialy

**Affiliations:** Department of Experimental and Clinical Physiology, Laboratory of Centre for Preclinical Research, Medical University of Warsaw, Warsaw, Poland

**Keywords:** ultrasonic vocalization, ultrasound separation, sexual behavior, 22-kHz, 50-kHz, rats

## Abstract

Ultrasonic vocalization (USV) is one of the measurable behavioral parameters of sociosexual interactions in rats. To precisely and accurately describe the neurobehavioral properties of USV and the potentially related specific emotional responsiveness of animals, we need to know which animals vocalize and what is their exact behavioral and physiological response. To this end, we modified the non-contact cage [non-contact erection model (NCE)] by adding a modification [vocalization-non-contact erection (VOC-NCE)] that makes it possible to assign emitted ultrasonic signals to a particular animal. Typically, the NCE cage consists of two compartments separated by perforated baffles. A male is placed in one section, and a receptive female is placed in the other section. This makes possible the accurate description of sexual parameters related to the cues controlled by the experimenter. In VOC-NCE, we completely separated the male USV from the female USV by three appropriately perforated baffles and located microphones combined with ultrasonic screening. We recorded emission in both typical bands, the so-called 22- and 50-kHz bands, with various subtypes, thus highlighting the utility of our protocol to investigate the sexual dimorphism of vocalization. Similar to the anticipatory model, we showed that emission can occur without acoustic feedback from concomitants during the VOC-NCE test. Therefore, we propose a relatively simple method for assigning individual vocalization. We discuss its usefulness and limitations in assessing vocal differentiation related to sexual parameters, adaptive changes during conditioning procedures, and further applications.

## Introduction

Ultrasonic vocalization (USV) emitted by rats has gained considerable acceptance in recent decades as an applicable parameter in neurobehavioral research (Burgdorf and Panksepp, 2006; Taracha et al., 2012; Potasiewicz et al., 2019; Caruso et al., 2020; Costa et al., 2021). The frequency spectrum of the main emitted signals ranges from 20 kHz to about 70 kHz. The commonly accepted division according to the dominance frequency divides them into two groups, the so-called 22- and 50-kHz bands (Sales and Pye, 1974; Wright et al., 2010; Wöhr and Schwarting, 2013; Brudzynski, 2015), differing in the accompanying neurobehavioral context of the emission and stimulation of the reward-related neuronal structure of the rat brain (Brudzynski, 2007).

During sociosexual interactions in rats (arousing and highly appetitive situations), we can observe a wide variety of ultrasounds with all subtypes in the 22- and 50-kHz bands (Barfield et al., 1979; Burgdorf et al., 2008; Seffer et al., 2014; Ågmo and Snoeren, 2015; Bialy et al., 2019a; Bogacki-Rychlik et al., 2021).

One of the most significant problems of USV analysis is assigning particular signals to the animal that emitted them. This is necessary for the standardization of USV as a parameter reflecting a specific physiological process taking place during intraindividual interactions. It could only then serve as being fully applicable in preclinical models with a reasonable translational potential. To date, methods allowing this type of distinction are based on an analysis of the anticipatory vocalization of each individual (Bialy et al., 2000; Bogacki-Rychlik et al., 2021), the devocalization of one of the animals (Thomas et al., 1981; White et al., 1990; Matochik et al., 1992; Kisko et al., 2015), the anesthetization of one of them (Maggio and Whitney, 1985), or the use of a complex system based on multisource recording combined with the application of mathematical methods (Sangiamo et al., 2020; Warren et al., 2020).

Each of these methods has specific limitations due to the invasiveness of the procedure, stimuli deprivation of the animals, or a methodology that is hard to apply.

We propose a relatively simple way to distinguish ultrasounds during the so-called vocalization-non-contact erection test (VOC-NCE) in the experiment described here. The non-contact erection test (NCE) and dedicated chamber cage were developed and refined by Benjamin D. Sachs (Sachs et al., 1994; Sachs, 1997). It made it possible to standardize non-contact erections as a behavioral parameter and use them to study mechanisms of sexual arousal through the selective and controlled deprivation of tactile, visual, or olfactory stimuli. The standard NCE procedure was carried out by placing two animals in two distinct compartments separated by three plexiglass partitions with specific perforations. Shifts in the level of perforations between the baffles provided olfactory stimuli without direct contact between the animals. Furthermore, the transparent versus opaque material of the partition gave some (or a reduced) visual stimuli. Also, the exchange of non-specific auditory stimuli (to reach the spectrum heard by humans) was possible due to the movements of the animals. By airflow regulation, the experimenter could control the level of olfactory stimuli perceived by the subjects (Sachs, 1997). Altogether, it provided an opportunity to measure the parameters of sociosexual behavior during the absence of direct tactile stimulation with a controlled level of other classes of stimuli.

We proposed an additional expansion of this method by adding the possibility of analyzing the USV parameter of a particular animal and thus correlate it with other sexual parameters of males and females. We obtained this selectivity by appropriately positioning two microphones (one in each compartment) and adding a silencing screen above the chambers. Consequently, we isolated the acoustic stream derived from each animal.

Moreover, due to the high research interest in USV during sociosexual interactions, we present in detail our investigative protocol, which is based on rat sexual behavior. We believe that sexual behavior across the rodent order, including typical laboratory models (i.e., mice, gerbils, hamsters, and rats) and their corresponding USV, is controlled by similar mechanisms but differs significantly in the detail. Thus, we hoped that the presented VOC-NCE chamber with the behavioral protocol would help to avoid misinterpretations of the results between different experimental approaches by implementing the possibility of controlling modalities combined with sexual parameters (general arousal, sexual arousal, sexual motivation).

Nevertheless, the primary aim of this model was to obtain a clear USV separation during sociosexual interactions.

## Materials and Equipment

### Apparatus

**FIGURE 1:**
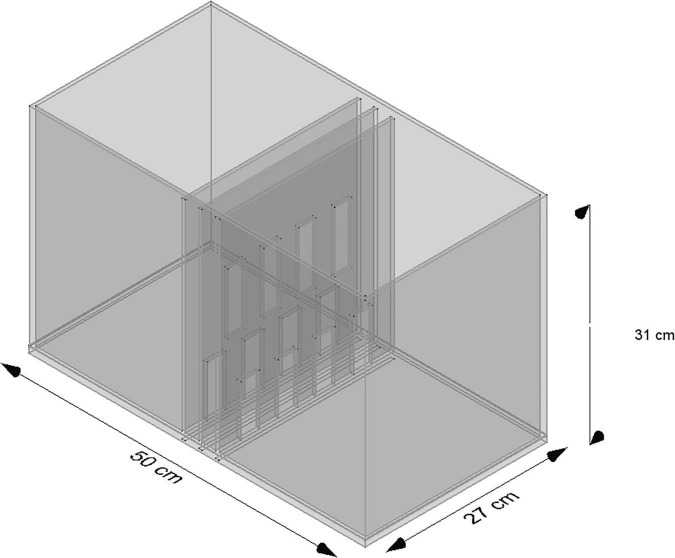
Scheme of the standard NCE cage. Two compartments are separated by three partitions, namely, two outer partitions with identical perforations and one middle partition with perforations shifted upward.

**FIGURE 2:**
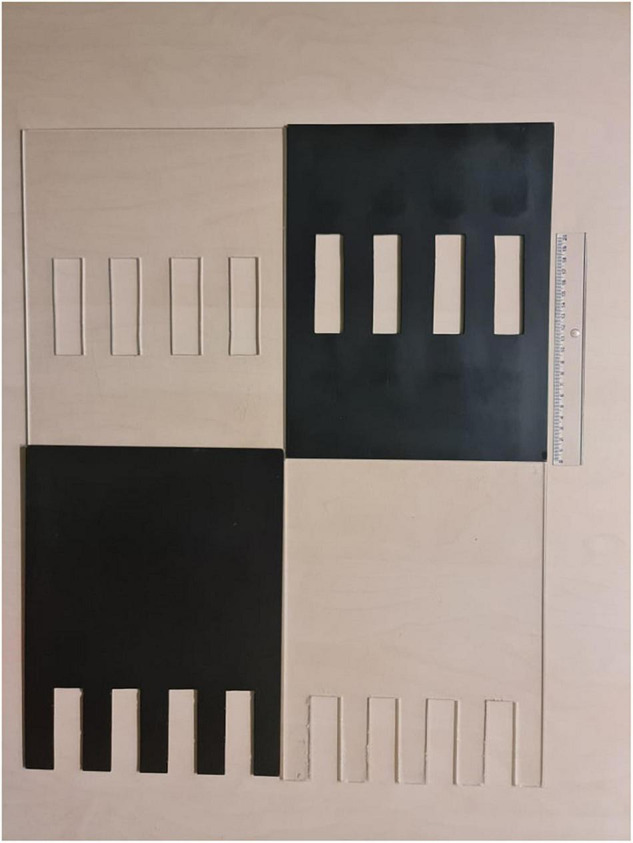
At the top of the figure are two middle partitions, and at the bottom are two outer partitions. The black opaque versions are alternatives to implement the deprivation of visual cues. The results presented in this study were obtained using the transparent version.

**FIGURE 3:**
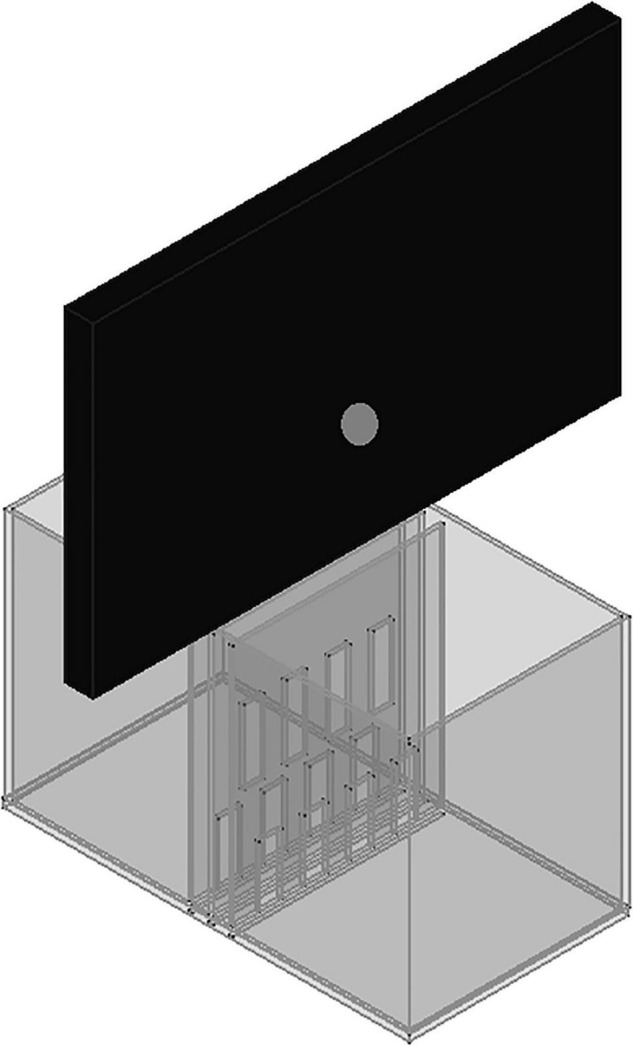
A scheme to illustrate the proposed expansion of the VOC-NCE cage with an acoustic screen.

**FIGURE 4:**
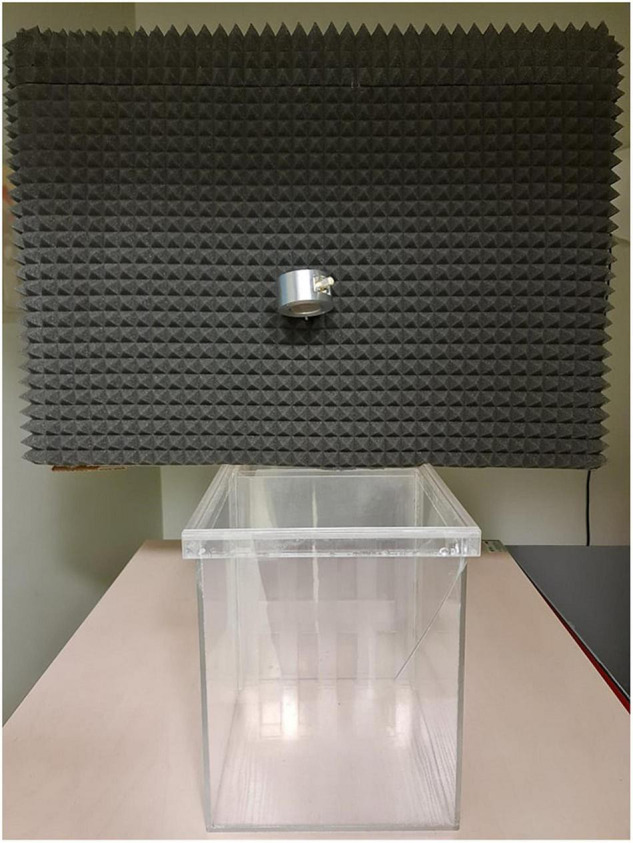
The photograph of the VOC-NCE located in the behavioral recording room (the frontal view). For clarity, cables have been unplugged from the microphones, and the covering grid was removed.

**FIGURE 5:**
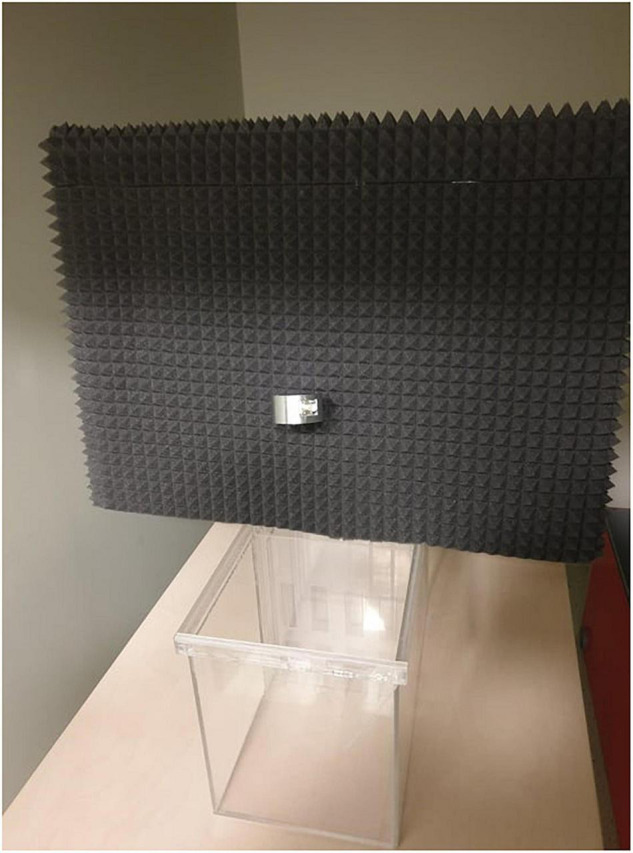
The photograph of the VOC-NCE located in the behavioral recording room (the oblique view). For clarity, cables have been unplugged from the microphones, and the covering grid was removed.

The experimental chamber, made of plexiglass, had total dimensions of 50 cm × 31 cm × 27 cm. For the VOC-NCE test, the long axis of the chamber was bisected in the middle of its length by a set of three transparent partitions placed 1 cm apart (Figures 1, 2). The two outer partitions had evenly spaced rectangular perforations (2.5 cm × 7.5 cm) cut out at the bottom. The middle partition had four similar-sized rectangular perforations (2.5 cm × 9 cm), aligned about 3 cm above the perforations of the outer ones (Figure 1). We covered the top of the compartments with a wire grid to prevent the rats from escaping during the experimental trials. To acoustically separate the recording compartments, we mounted an acoustic screen (72 cm × 52 cm × 10 cm) in the central part of the lid, just above the three dividers. The microphones were attached 50 cm above the ground, one on each side, exactly opposite each other (Figures 3–5).

### Technical Assumption

In the experiment, we used a cage constructed of poly (methyl methacrylate) (PMMA), also known as plexiglass. The choice of material was dictated by its properties such as low weight, transparency (as PMMA transmits up to 94% of visible light) (Martín-de León et al., 2019), high flexibility (good resistance to cracking compared with polystyrene as used in some systems), and ease of cleaning even at high temperatures (Ali et al., 2015). However, in our experiment, the most important features are related to its sound insulation properties. In this context, PMMA is used in environmental engineering as sound walls to reduce industrial noise (Garai and Guidorzi, 2000), acoustic screens in recording studios, and commercially available isolation systems for animals (such as IVC). In the presented VOC-NCE system, two compartments were separated by three partitions made of plexiglass. This made it possible to obtain sufficient ultrasonic isolation due to the high acoustic impedance of the material. The main frequency range of the USV of the rats is between 20 kHz to about 70 kHz, so the wave energy is relatively low (comparable with the human audible sound wave range). We decided to leave open each compartment at the top and isolate them by the acoustic splitter screen (covered with polyurethane soundproofing foam). The open construction made it possible to avoid sound artifacts in the recordings generated due to multiple wave reflections. Apart from the ability to reflect, plexiglass has a significant sound absorption parameter, so we could probably create an acoustic shadow in the middle part of the cage (between the outer partitions). The floor of the VOC-NCE cage was not covered by bedding to reduce ultrasonic artifacts.

### Methods

#### Animals and Behavioral Procedures

The subjects of this study were 6-month-old Long-Evans rats (eight males and eight females, weighted 480–570 g and 280–400 g, respectively). The animals were purchased from the Department of Experimental Medicine, the Medical University of Silesia, Katowice. The males and females were housed in separate rooms, with two animals per standard laboratory cage (55.6 cm × 33.4 cm × 19.5 cm, Animalab type IV). Chow and water were freely available. All cages were provided with wood shavings and dedicated plastic tubes as enrichment. The rats were maintained on a 12-h light-dark cycle (lights switched off at 9:00 p.m.) at a temperature around 22°C. All of the behavioral tests were conducted between 13:00 and 17:00 during the dark phase of the light-dark cycle. The experimental room was illuminated primarily by infrared light, with a total intensity of 25 lux.

The females were bilaterally ovariectomized [under intraperitoneal (IP) injection of ketamine 10 mg/100 g with xylazine 1 mg/100 g] before the experiment and then convalesced for 2 weeks (with analgetic and anti-inflammatory support). Estrus was hormonally induced with a single injection of estradiol benzoate [50 μg/rat subcutaneously (s.c.), Sigma, 48–72 h before the trial] and progesterone (500 μg/rat s.c., Sigma, 4–8 h before the trial). Hormones were dissolved in sesame oil and administered in 0.05 ml doses per individual. The induction was repeated no more than once a week and at least once every 2 weeks. The animals were habituated to the experimenters, to all behavioral procedures, and to the chamber in all versions (for the copulatory and VOC-NCE test). Before the trial phase, all of the animals acquired sexual experience during four preceding training sessions. Training copulatory sessions were performed in the experimental VOC-NCE chamber with the partitions removed. The male was placed for 5 min for acclimation (anticipatory phase), and then an estrus female was introduced for 25 min or up to the first intromission after the first ejaculation.

During the experimental trials, a male was placed in the first compartment for 5 min alone (anticipatory phase) and the recording equipment was switched on. After this time, a female was placed in the second compartment, and the recording process continued for the next 20 min.

### Ultrasonic Recording and Analysis

All behavioral procedures were performed in two adjoining rooms. The recording room with the VOC-NCE cage and microphones and infrared camera was acoustically and visually isolated from the observers. A second room was used for observation, registration (computer with hardware), and further analysis. The rats were placed in the recording room during all of the trials while the experimenters stayed in the other room.

This separation isolated unwanted stimuli and reduced background noise generated by the electronic equipment and human activity (to maintain an optimal acoustic environment). The USV was recorded with a Sonotrack System (Sonotrack by Metris ver. 2.2) dedicated to this purpose, which enables recording on up to four independent parallel channels. The system consists of ultrasonic microphones (frequency range 15--125 kHz), with fully compatible hardware and software^1^. The microphones were connected by two identical cables, identical in all conductive parameters. All USV tracks were analyzed and compared manually. After the visual analysis based on spectrogram pictures, analog time windows were listened to, to exclude potential low-energy vestigial ultrasonic leaks.

## Results

**FIGURE 6:**
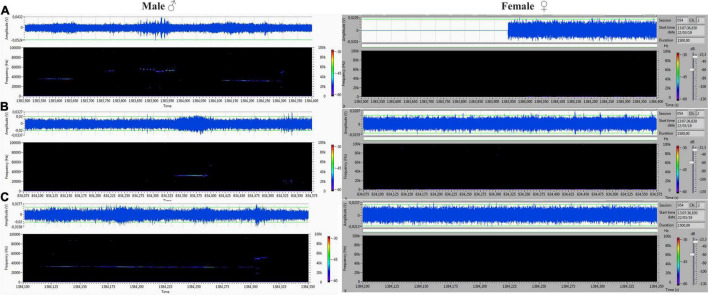
**(A,C)** Different forms of 22-kHz specific for VOC-NCE: composite forms with different lengths of 22-kHz signal with co-occurring higher frequencies. **(B)** Short form of 22 kHz. All of them were emitted by males, and analogical parts of the female‘s spectrogram were blank.

**FIGURE 7:**
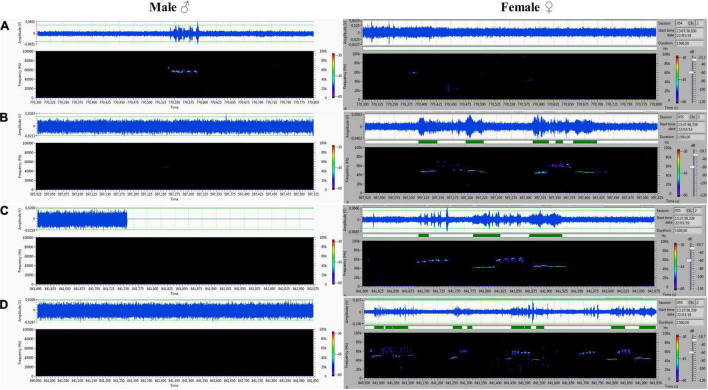
**(A)** Frequency-modulated non-trill (complex) emitted by the male. Female emitted two forms of short type. **(B–D)** Very intensive, frequency-modulated USV with trills and composite clusters of signals emitted by the female. The male was silent.

**FIGURE 8:**
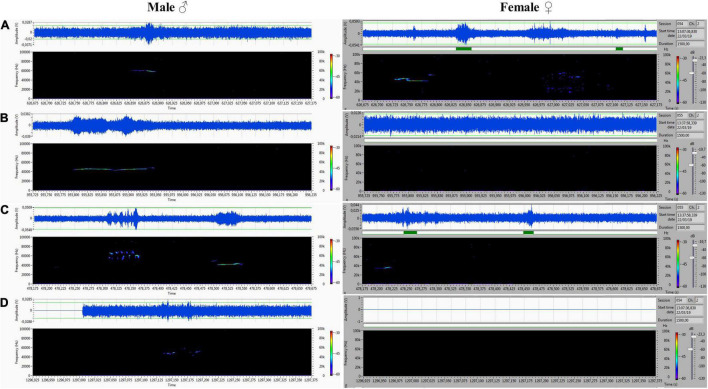
**(A–D)** Various forms included flat-type, multistep, and step down on the male’s side On the female’s side, the ultrasonic spectrum is distinctly different. All ultrasounds can be unambiguously assigned to the emitter.

**FIGURE 9:**
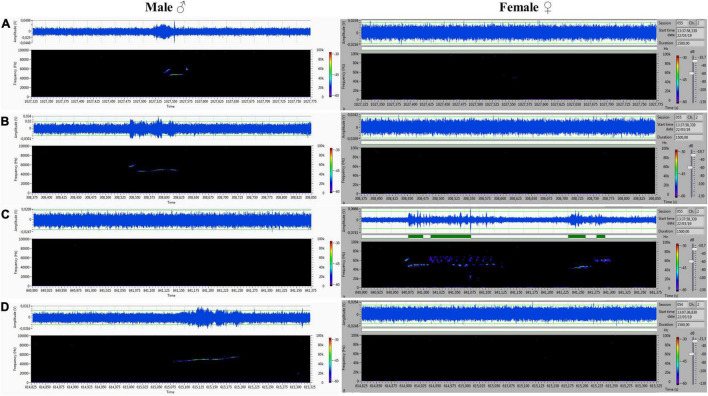
**(A–D)** Another set of ultrasounds with split type, upward, and step down on the male’s side, and frequency-modulated trills on the female’s side.

The acoustic isolation was sufficient to separate all emitted ultrasonic calls and assign them to each animal. We analyzed the records from the male (channel 1) and female (channel 2) compartments obtained during non-contact sessions. Vocalizations were very intensive (hundreds of particular signals per track), and both males and females vocalized in all of the main types of ultrasounds. We presented some arbitrarily chosen samples of ultrasounds from two random records (Figures 6–9). The only criterion for selection was our intention to demonstrate various types in both 50- and 22-kHz frequencies. The visible color differentiation of the software was intentionally applied to improve the overall clarity. The timeline of the corresponding spectrograms was precisely adjusted to each other to reveal sufficient separation.

## Discussion

Our results indicate that the proposed VOC-NCE procedure is sufficient to separate vocalizations emitted by two individuals during sociosexual interaction. We have demonstrated that partitions with appropriately located perforations, combined with simple acoustic isolations, are sufficient to entirely reduce the ultrasonic stream while maintaining a flow of other stimuli. In the conducted sexual vocal-non-contact tests, emissions of USV were very intensive in the situation with reduced acoustic feedback, which implies the dominative role of cues other than auditory in initiating and maintaining USV. Therefore, this relatively simple model provides an opportunity to prioritize incentive modalities, crucial for emission during encounters. Moreover, it makes it possible to describe sex-dependent features in the spectrograms obtained due to reliable matching to the male or female. Furthermore, the presented separation should be useful for assigning an individual USV profile to other physiological parameters occurring during sexual behavior.

### Expanding the NCE Test

In the classical procedure presented by Benjamin D. Sachs, the visual analysis of behavior enables the parameterization of sexual arousal (by latency to first non-contact erection as well as the number of non-contact erections) (Sachs et al., 1994; Sachs, 1997), sexual preferences (Kondo and Hayashi, 2021), sociosexual motivation (latency to start and duration of perforation sniffing), and general arousal (number of rearing, total locomotor activity). The experimental non-contact chamber provides visual contact (when the baffles are transparent), exchange of olfactory stimuli due to perforations (olfactory cues could also be reduced or enhanced by pumping air in or out), and non-specific acoustic cues as a result of animal movements (mainly in the human audible range). The previous experiments have shown that olfactory cues are sufficient and the most important to evoke non-contact erections (Sachs, 1997; Kondo et al., 1999) in mechanisms related to the medial amygdala activity (Bialy and Sachs, 2002; Kondo and Sachs, 2002; Bialy et al., 2011). Other stimuli appear to be less relevant in inducing a non-contact erection and sexual arousal in rats (Sachs, 1997). We can also modify the level of sexual arousal *via* the hormonal status of the female or male (Sachs et al., 1994; Manzo et al., 1999; Fernández-Vargas, 2018). Taken together, VOC-NCE gives opportunities to investigate USV during sociosexual activity in a more precise and selective established cue/sensory environment.

### Ultrasonic Vocalization During VOC-NCE, Anticipatory, Copulation, and Postejaculatory Period: Behavioral Protocols

#### USV in the VOC-NCE Test

**FIGURE 10:**
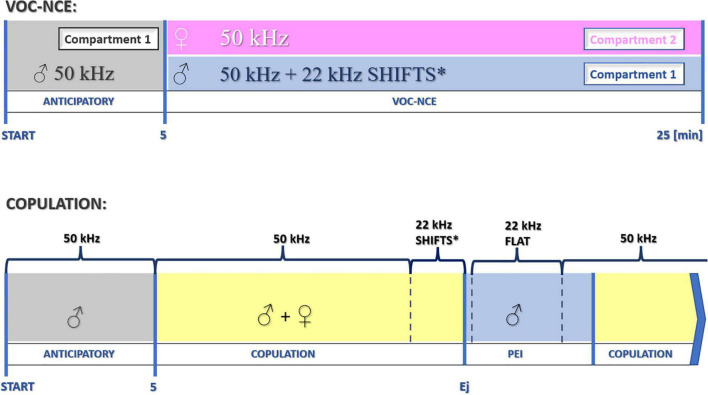
Schematic protocol for the investigation of USV during particular phases of sociosexual interactions. *22-kHz shifts in the late phase of copulation occur relatively rarely. After the dominant flat-type postejaculatory 22-kHz signal, some forms of modulation could also be observed (end phase of 22-kHz postejaculatory vocalization, not depicted in the figure). Ej, ejaculation.

We started to measure the vocalization phase of the VOC-NCE test after 5 min of the anticipatory period with the introduction of the female. The duration of the anticipatory phase is determined during the preceding sexual training procedure. However, 5 min is commonly accepted due to optimal acclimation/conditioning properties during sexual behavior (Larsson, 1956; Bialy et al., 2000). In this and previous experiments, we end the NCE sessions after 20 min with a female (25 min in total, refer to Figure 10).

Previously, we found vocalizations specific to the NCE test in the so-called 22-kHz band. This variation included frequency-modulated signals and assembled types with coexisted prefixes or suffixes (Bialy et al., 2019a). By applying the VOC-NCE expansion, we have accurately identified particular emitters, in this case, by separating the ultrasound from the male and female. Thus, it probably could be functional in assessing some sexual dimorphic traits in spectrograms due to recent interest in this field (for a detailed review, refer to Lenell et al., 2021).

During 20 min of VOC-NCE, the USV of both male and female in physiological conditions should be abundant, with domination of the 50-kHz signals in the recordings. In this phase, we observed many high-frequency-modulated signals (trill-like types) co-occurring with all other subtypes of the 50-kHz group. These subtypes (trills and flat-trill) can be considered as additional markers of estrus occurrence. Our observations are consistent with the results of Barfield and Thomas (1986), which showed estrus clustered and a modulated type of USV. Similarly, Gerson et al. (2019) showed that direct clitoral stimulation elicits trill and flat-trill subtypes, and hormones significantly augment their emission. We demonstrate in Figures 7, 9 this characteristic pattern of estrus (aroused) female vocalization with highly modulated 50-kHz signals combined in clusters. This type of USV is relatively rare in the recordings of males during non-contact. In contrast, in the male spectrograms, we observed characteristics for the non-contact test variant of a 22-kHz, i.e., the 22-kHz shift-subtype consisting of a 22-kHz call combined with a 50-kHz prefix or suffix. They seem to reflect a fluctuation of arousal that can be interpreted as an emotional state related to sexual frustration. We have described these subtypes in detail previously (Bialy et al., 2019a). Different patterns of frequency-modulated 22-kHz USVs have also been demonstrated in many other neurobehavioral contexts (Miczek and Van Der Poel, 1991; Vivian and Miczek, 1993; Karwicka et al., 2021).

The implied question arises about the possibility of experimentally controlling the frustration levels using VOC-NCE. Potentially, it could be achieved with the implantation of removable (openable) partitions, which would allow direct contact between the male and female after a fixed time of the non-contact procedure (Pfaus et al., 1990; similarly to Mas et al., 1990, 1995; Damsma et al., 1992; Lorrain et al., 1997, 1999). In such an experimental setup, the acquisition of sexual experience should reduce frustration effects *via* the conditioning processes during trials (Pfaus et al., 2001).

Overall, based on the presented VOC-NCE model, it is possible to conduct a quantitative and qualitative analysis of the 22- and 50-kHz bands to construct individual USV profiles, which can be used as an additional parameter to describe behavioral changes during sexual activity encounters.

During sexual behavior, we found that male rats in different phases of sexual behavior (anticipatory, postejaculatory period, and NCE test) display a specific pattern of vocalizations with dominant USV subtypes (Bialy et al., 2019a; Bogacki-Rychlik et al., 2021). This suggests that specific central responsiveness (emotional/motivational/arousal) influences the USV pattern. Therefore, we discuss in more detail these phases and compare them to the NCE and VOC-NCE tests.

#### Anticipatory Pre-Contact Vocalization

Anticipatory sexual behavior makes possible the analysis of individual rat USVs. During our experiments, males vocalize frequently, and such vocalizations are related to emotional memory, sexual motivation, conditioning to odor and background cues, sexual experience, and level of general arousal, but not sexual arousal *per se* (Bialy et al., 2019b). The total number of anticipatory precontact 50-kHz vocalizations (PVs) positively correlates with the acquisition of sexual experience, rewarding the value of previous sociosexual contact (Bialy et al., 2000), and the level of sexual motivation (Bogacki-Rychlik et al., 2021). Contrary to the total number of PVs, the pattern of vocalizations measured by the percentage of different subtypes seems to be stable and characteristic of anticipatory behavior with dominant complex/composite and flat calls (Bogacki-Rychlik et al., 2021). In the anticipatory phase, 22-kHz signals occur very rarely, and if they do then in short and unmodulated forms. Also, in this phase, erections of the penis, a sign of sexual arousal, are not observed in the standard 5-min period, supporting the thesis that PVs reflect sexual motivation and general arousal but not sexual arousal *per se*.

#### USV During Copulation

Copulation of male rats is a relatively complex but well-parameterized behavior (Sachs and Barfield, 1976; Sachs, 1978; Bialy et al., 2000, 2019b; Hull and Rodríguez-Manzo, 2017). During copulation, many ultrasounds are emitted by both male and female, mostly at 50 kHz. The female vocalizes intensively during solicitation (darting, hopping, and ear wiggling), and the level of USV depends on the hormonal status. The male vocalizes during an approach to the female, especially before investigation and mounting (Barfield and Thomas, 1986). This USV significantly increases when having a problem with achieving intromissions and ejaculation, thus suggesting the significant effect on USV production of some frustration and enhanced general arousal rather than sexual arousal and sexual motivation *per se* (McIntosh and Barfield, 1980; Bialy et al., 1996). The role of the male and female USV in the coordination of copulation was investigated by using devocalized animals. A female exposed to a devocalized male displays less intensive soliciting behavior and more frequently moves out during intromission or ejaculation (Thomas et al., 1981). Such results suggest some coordinator role of the USV in the sexual interaction, although the effect of female or male devocalizations on copulatory parameters is not statistically significant (Ågmo and Snoeren, 2015).

#### USV During the Postejaculatory Interval

The PEI is the period between ejaculation and the next cycle of copulation, first mounting, or intromission after a refractory period (Sachs, 1978; Hull and Rodríguez-Manzo, 2017). In the USV spectrograms of males, there is a domination of specific long-lasting 22-kHz calls with a very stable frequency (Barfield and Geyer, 1972; Bialy et al., 2019a). Postejaculatory vocalizations occur as the effect of an abrupt decrease in the arousal and motivation level with sleep-like patterns in EEG (Kurtz and Adler, 1973; Barfield and Geyer, 1975) and thus seem to reflect the relaxation state of the male after ejaculation (Bialy et al., 2016). The intensity of 22-kHz postejaculatory (latency to start and duration time) depends on the presence of the female (Sachs and Bialy, 2000). However, the crucial cues responsible for this reaction are not clear, but an enhanced level of anxiety inhibits this type of vocalization (Bialy et al., 2016). The 50-kHz types in PEI are uncommon when the 22-kHz signal occurs and are usually observed after it ends. Thus, their presence indicates increasing sexual motivation before the next copulation cycle (Figure 10).

Overall, we can distinguish behavioral parameters during PEI, which imply the time of enhancement of general arousal, sexual arousal, and sexual motivation (Sachs and Bialy, 2000; Bialy et al., 2019b), and the presented forms of USV are some of them.

#### VOC-NCE vs. Anticipatory PV, Copulatory and Postejaculatory USV

The VOC-NCE model presented here, as well as anticipatory PV, is a non-invasive method to analyze individual USV. The anticipatory PV model is convenient for investigating the male USV related to sexual motivation and general arousal (Bialy et al., 2019b). In both situations, the tactile and taste cues from the female are absent. The VOC-NCE model gives an additional opportunity, that is, we can investigate male and female USV individually in a state of the enhanced level of sexual arousal (together with general arousal and sexual motivation). In male rats, sexual arousal is evoked by odors from female urine and the body (Sachs, 1997; Kondo et al., 1999), and in estrus females by hormones, male odors, and the presence of males (Barfield and Thomas, 1986).

Both VOC-NCE and copulation trigger USV, and in both situations enhanced levels of sexual arousal, general arousal, and sexual motivation can be observed. In the VOC-NCE test, direct contact with tactile stimulation essential in copulation is absent (nor taste cues). There is a lack of consummatory elements of sexual interaction, namely, intromission, ejaculation, and hypothetically rewarding mounting. Thus, the rewarding and (eventually) some aversive aspects specific to copulation are not present. Also, we do not observe a relaxation state typically observed after ejaculation, and as an implication, a PEI does not occur (we can observe a rather gradual than a rapid decrease of arousal levels). These differences have a reflection in the changes in the 22-kHz USV mentioned above.

Overall, the VOC-NCE protocol could be used as an expansion of the standard anticipatory PV + copulation, providing an opportunity to obtain individual USV profiles (for summarization of differences between models, refer to Table 1 and Figure 10).

**TABLE 1:** Summary of general arousal (GA), sexual arousal (SA), sexual motivation (SM), and genital tactile (GT) cues in different sexual situations.

	GA	SA	SM	GT
PV (anticipatory)	H	L	H	-
Copulation	H	H	H	H
PEI	I	I	I	L (genital grooming)
VOC-NCE	H	H	H	L (genital grooming)

*H, high; L, low; I, increasing from low to high.*

## Conclusion

Our modification of the non-contact test makes it possible to separate USV emitted by a pair of animals. It provides an opportunity to regulate the intensity of all modalities, including the ultrasonic stream, perceived during sociosexual interactions, and verify the influence of this change on USV profiles. We proposed the VOC-NCE cage as a new, non-invasive tool, expanding the classical sociosexual protocol (anticipatory plus copulatory). This makes it possible to describe changes in arousal and motivation during conditioned procedures by noticing differences in the USV, which should be useful in investigations of sociosexual behavior.

### Additional Applications

Our results indicate that the ultrasound vocalization has been emitted intensively with significantly reduced ultrasonic feedback from another animal. In such a view, USV could be considered a behavioral reflex resulting from the activation of central autonomic nuclei (e.g., RVLM/CVLM, NTS, nucleus ambiguus) with effector response of the laryngeal and expiratory muscles (Hernandez-Miranda et al., 2017). Thus, it is comparable to the objective component of emotional response and has a non-discrete informational character perceived by concomitants. During sexual behavior, there is a remarkable parallel increase in sympathetic and parasympathetic nervous system activity. Therefore, the vocalization corresponding to this behavior is a reflection of this autonomic equilibrium, and as such, can be applied as a preclinical parameter. It is extremely valuable, especially if we combine it with other autonomic parameters detected by telemetric systems, e.g., heart rate (Filipkowski et al., 2017; Olszyński et al., 2021) and arterial blood pressure (Vianna et al., 2008).

Based on our set of experiments, we tried to present an interesting differentiation in the 22-kHz signals. The results should be considered as an argument for separating them into distinct subgroups, namely, the modulated types of NCE procedure, and the flat, long-lasting postejaculatory. They correspond to very different neurobehavioral contexts and clustering them together with a “negative-feeling” reference seems to be inappropriate. Additionally, the VOC-NCE model might help to answer the question of the communication role of USV *via* influence on the behavior of the concomitant animal and, with that, assess its communicative function. There is a recurring issue about how much of this vocalization is communicative (Sales and Pye, 1974). To be more precise, what is the proportion between phylogenetically old, non-specific stem-derived motoric reactions of the laryngeal muscles as a result of fluctuating arousal (Delius, 1970; Bell, 1974; Pfaff, 2017) and the evolutionary younger, sociobiological messages controlled by emitters reflecting distinct emotional pathways (Panksepp, 2005; Brudzynski, 2013; Wöhr et al., 2021).

Although this system is dedicated to highly appetitive sociosexual interaction, it might be applicable in other behavioral contexts. For example, during social male-male or female-female interactions, the USV profile can correspond with same-sex preference (Van de Poll and Van Dis, 1979; Olvera-Hernández et al., 2015), social buffering, anxious-type behavior, or agonistic behavior/aggressive reactions.

Additionally, this model may be seen as useful in pharmacological and neurobehavioral experiments to evaluate the potential influence of various agents on sexual parameters, which includes selective serotonin reuptake inhibitors, serotonin-norepinephrine reuptake inhibitors, antipsychotic drugs, other 5-HTr modulators, dopamine receptor agents, autonomic nervous system agents, phosphodiesterase inhibitors, nitrates, other types of endogenic substances, and toxic factors (Olivier et al., 2006, 2017, 2022; Lin et al., 2015; Sanna et al., 2015; Chiang and Park, 2020; Esquivel-Franco et al., 2020; Kermath et al., 2022). Similarly, it could be useful in investigations of emotional responses, in addition to models of erectile dysfunction or premature ejaculation (Hull et al., 1994; Sachs, 2000; Waldinger and Olivier, 2005; Olayo-Lortia et al., 2014).

## Limitations

In a few cases of singular ultrasounds (not presented here), some form of vocal artifacts was recorded, probably from the concomitant. However, appropriate matching was easy to apply due to the high difference in signal energy. We decided to leave this vestigial ultrasonic leak as an additional non-specific stimulus (blurred and weak, amorphic shapes of ultrasounds cannot be considered to be a distinct communicative signal). Nevertheless, it has to be emphasized that the isolation was nearly total, and the residual leak could be reduced by a slight change in perforation size or adjusting the threshold points in the recording settings. Other limitations stemmed directly from the non-contact procedure and have already been discussed.

## Data Availability Statement

The original contributions presented in the study are included in the article/supplementary material, further inquiries can be directed to the corresponding author/s.

## Ethics Statement

The animal study was reviewed and approved by Local Ethical Committee in Warsaw.

## Author Contributions

WB-R and MB designed and performed the experiments, analyzed the data, and prepared the manuscript. AW analyzed the USV data and prepared the manuscript. All authors contributed to the article and approved the submitted version.

## Conflict of Interest

The authors declare that the research was conducted in the absence of any commercial or financial relationships that could be construed as a potential conflict of interest.

## Publisher’s Note

All claims expressed in this article are solely those of the authors and do not necessarily represent those of their affiliated organizations, or those of the publisher, the editors and the reviewers. Any product that may be evaluated in this article, or claim that may be made by its manufacturer, is not guaranteed or endorsed by the publisher.

## Footnotes

1 refer to https://www.metris.nl/en/products/sonotrack/sonotrack_specs/forspecifications.
